# Data Independent Acquisition Mass Spectrometry Enhanced Personalized Glycosylation Profiling of Haptoglobin in Hepatocellular Carcinoma

**DOI:** 10.1021/acs.jproteome.4c00227

**Published:** 2024-07-12

**Authors:** Tiara Pradita, Yi-Ju Chen, Tung-Hung Su, Kun-Hao Chang, Pei-Jer Chen, Yu-Ju Chen

**Affiliations:** † Institute of Chemistry, Academia Sinica, Taipei 115, Taiwan; ‡ Sustainable Chemical Science and Technology, Taiwan International Graduate Program, Academia Sinica, Taipei 115, Taiwan; § Department of Applied Chemistry, National Yang Ming Chiao Tung University, Hsinchu 300, Taiwan; ∥ Division of Gastroenterology and Hepatology, Department of Internal Medicine, National Taiwan University Hospital, Taipei 100, Taiwan; ⊥ Hepatitis Research Center, National Taiwan University Hospital, Taipei 100, Taiwan; # Molecular Science and Technology Program, Taiwan International Graduate Program, Academia Sinica, Taipei 115, Taiwan; ○ Department of Chemistry, National Tsing-Hua University, Hsinchu 300, Taiwan; ◆ Graduate Institute of Clinical Medicine, National Taiwan University College of Medicine, Taipei 100, Taiwan; ∇ Department of Medical Research, National Taiwan University Hospital, Taipei 100, Taiwan; ◧ Department of Chemistry, National Taiwan University, Taipei 106, Taiwan

**Keywords:** data-independent acquisition (DIA), glycoprotein, haptoglobin, hepatocellular carcinoma (HCC)

## Abstract

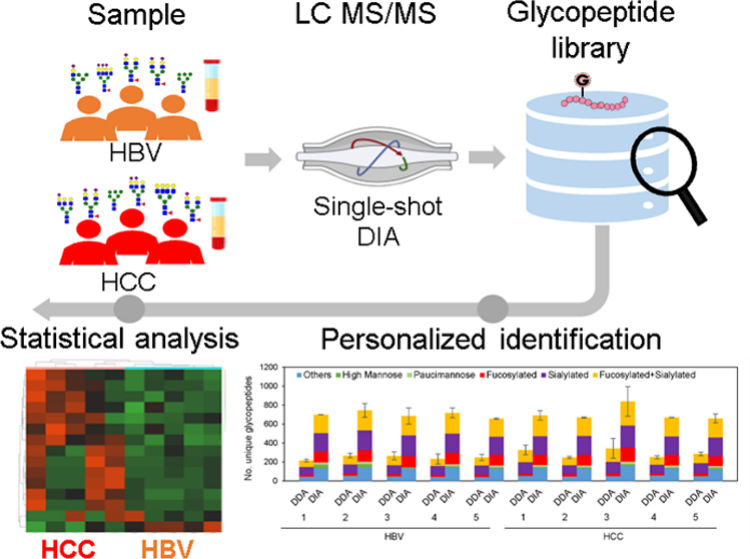

Aberrant glycosylation has gained significant interest for biomarker discovery. However, low detectability, complex glycan structures, and heterogeneity present challenges in glycoprotein assay development. Using haptoglobin (Hp) as a model, we developed an integrated platform combining functionalized magnetic nanoparticles and zwitterionic hydrophilic interaction liquid chromatography (ZIC-HILIC) for highly specific glycopeptide enrichment, followed by a data-independent acquisition (DIA) strategy to establish a deep cancer-specific Hp-glycosylation profile in hepatitis B virus (HBV, *n* = 5) and hepatocellular carcinoma (HCC, *n* = 5) patients. The DIA strategy established one of the deepest Hp-glycosylation landscapes (1029 glycopeptides, 130 glycans) across serum samples, including 54 glycopeptides exclusively detected in HCC patients. Additionally, single-shot DIA searches against a DIA-based spectral library outperformed the DDA approach by 2–3-fold glycopeptide coverage across patients. Among the four N-glycan sites on Hp (N-184, N-207, N-211, N-241), the total glycan type distribution revealed significantly enhanced detection of combined fucosylated-sialylated glycans, which were the most dominant glycoforms identified in HCC patients. Quantitation analysis revealed 48 glycopeptides significantly enriched in HCC (*p* < 0.05), including a hybrid monosialylated triantennary glycopeptide on the N-184 site with nearly none-to-all elevation to differentiate HCC from the HBV group (HCC/HBV ratio: 2462 ± 766, *p* < 0.05). In summary, DIA-MS presents an unbiased and comprehensive alternative for targeted glycoproteomics to guide discovery and validation of glyco-biomarkers.

## Introduction

Advancement in glycoproteomics provides a powerful tool to uncover the complex and dynamic glycosylation, site-specific glycans, and carrier glycoproteins. Delineation of the altered structure-phenotype provides insight on their functional consequences in health and disease.^1^ Alterations in the glycoprotein expression or its glycan structures have been reported to be key cellular mechanisms regulating cell signaling and communication, tumor cell invasion, cell–matrix interactions, tumor angiogenesis, immune modulation, and metastasis in cancer,^2^ such as liver cancer,^3^ pancreatic cancer,^4^ and lung cancer.^5^ Targeting altered protein glycosylation may inspire development of biomarkers for early detection and prognosis. In the example of hepatocellular carcinoma (HCC), the fifth most common cause of cancer worldwide and top leading cause of cancer death,^6^ upregulation of glycosyltransferases has been found to be associated with progression from liver diseases to HCC.^7−9^ However, due to the low stoichiometry, highly complex structure and heterogeneity of site-specific glycans across glycosites and relatively low detectability of intact glycopeptides, full characterization of glycoproteome still presents challenging tasks.^10^

Serum is an important source of biomarkers because the liver synthesizes and secretes a significant portion of blood proteins, potentially containing molecular indicators that reflect the development of liver disease.^11,12^ The exploration of glycosylation aberrations in serum glycoproteins associated with cancer offers a promising approach for identifying specific and sensitive biomarkers to improve current diagnosis. In HCC, early diagnosis remains an unmet need to monitor the development of HCC from risk factors, including different etiologies, chronic hepatitis B virus (HBV), hepatitis C virus, alcoholic liver disease, and nonalcoholic fatty liver disease, and specifically HBV infection, which is associated with more than 70% of HCC worldwide.^13,14^ The lifetime incidence of HCC in individuals with chronic HBV infection is reported to be approximately 10–25%.^15^ Comparatively, the global prevalence of HBV among patients with cirrhosis is around 42%.^16^ However, it is also important to note that most cases of HBV-associated HCC occur in cirrhotic liver disease, present in 70–90% of cases.^13,14^ Furthermore, quantitation of FDA approved HCC biomarker alpha-fetoprotein (AFP) has limited diagnostic sensitivity due to elevated expression in only 60–70% of HCC cases as well as low specificity for false positive diagnosis in other liver diseases like chronic hepatitis and cirrhosis.^17,18^

In recent years, serum haptoglobin (Hp), synthesized and secreted into the blood from the liver for binding free hemoglobin (Hb) to prevent kidney damage caused by released iron, has gained increasing interest due to its potential as a biomarker for liver diseases.^19−23^ Hp contains four N-glycosylation sites (N-184, N-207, N-211, N-241), and its N-glycan classes found in haptoglobin include complex, hybrid, and high-mannose structures. These classes are distinguished by their branching patterns and presence of modifications of sialic acid and fucose. Alterations in the glycosylation patterns of haptoglobin, such as aberrant fucosylation, sialylation, and branching are associated with various diseases, including cancer.^24^ Zhu et al. reported significant elevation of five N-glycopeptides at sites N-184 and N-241 during the progression from nonalcoholic steatohepatitis (NASH) cirrhosis to HCC (*p* < 0.05); specifically, N-glycopeptides bearing a monofucosylated triantennary glycan (A3G3F1S3) exhibit the best diagnostic performance in early detection of NASH-related HCC.^22^ The most recent work by Kohansal-Nodehi et al. discovered four Hp N-glycopeptides that demonstrate high potential for detecting early stage HCC (AUC > 80%), specifically monofucosylated-tetra-sialylated tetraantennary glycan at the N-207 site (AUC > 90%, sensitivity > 86%, and specificity > 75%).^23^ These DDA-based profiling results revealed the heterogeneity of the Hp glycosylated variants as a promising glyco-biomarker for HCC.

The most common approach for glycoproteomic profiling uses the data-dependent acquisition (DDA) method. However, a major challenge of the DDA method is the semistochastic identifications that cause missing values, poor reproducibility, and quantitation.^10^ Its performance for glycopeptide identification is significantly influenced by the heterogeneity, low abundance, and low detectability in LC-MS/MS. To overcome ion suppression from unmodified peptides, extensive enrichment and fractionation are often required to achieve a deep coverage which, in return, demands large sample quantities.^25^ Alternatively, data-independent acquisition (DIA) offers unbiased fragmentation of all precursor ions within a user-defined *m*/*z* range (usually 10–20 Da) and gains increasing popularity to improve detection sensitivity and quantification accuracy. At single glycoprotein level, pioneering DIA work by Pan et al. demonstrated improved site-specific glycan identification from IgM spiked in yeast lysate compared to DDA.^25^ Lin et al. developed a targeted DIA workflow that identified 41 glycoproteins from HILIC-enriched human plasma.^26^ Yang et al. reported a GproDIA pipeline that applies peptide-centric DIA analysis combined with a sample-specific library (SSL) and lab repository-scale spectral library (LRL) to improve 14% and 35% more glycan identifications in human serum, respectively.^27^ Ye et al. reported O-glycopeptides libraries (2076 O-glycoproteins and 5 most common core O-glycan structures), which facilitated identification of 269 O-glycopeptides (159 glycoproteins) from six human serum samples, even without glycopeptide enrichment.^28^ Chang et. al compared the DIA and DDA mass spectrometry methods in the context of SARS-CoV-2 and vaccine development. They found that DIA (183 glycosite, 458 glycoforms) identified more glycoforms among all glycosylation sites than DDA (73 glycosite, 118 glycoforms).^29^ Without glycopeptide enrichment, Sanda et al. reported the GP-SWATH workflow allowing quantitative comparison of 130 glycoforms of 28 glycopeptides from plasma of liver cirrhosis patients.^30^ More recently, Dong et al. reported the use of 100 isolation windows for DIA combined with a serum spectral library (1123 unique glycopeptides), which offers quantification for 620 glycopeptides in human serum.^31^ These encouraging reports suggest DIA may improve identification coverage for glyco-biomarker, yet its application to biomarker discovery remains underexplored.

We previously established DIA-MS to demonstrate enhanced profiling coverage and sensitivity in large-scale proteomics and phosphoproteomics^32^ or at the nanoscale and single cell level.^33,34^ Due to the much greater challenges of structure complexity and highly complex DIA-MS2 spectra in the glycoproteomics, we use Hp protein to serve as the first model study for method development and validation. Herein, we reported a DIA-based platform to demonstrate its enhanced performance and unique features for identification and quantitation. Specifically, the platform integrated two step enrichments, including hemoglobin conjugated magnetic nanoparticles (Hb@MNPs) for rapid Hp enrichment from serum and zwitterionic hydrophilic interaction liquid chromatography (ZIC-HILIC) Stage-Tips for glycopeptide enrichment, a high quality Hp glycopeptide spectral library, followed by single shot DIA-HCD MS/MS analysis. By systematic comparison with the performance of DDA, DIA shows higher recovery of glycopeptide toward comprehensive profiling on glycopeptides and complex types of glycan structures. The pipeline was applied to establish personalized Hp glycosylation profiles across patients with HCC and HBV. Our results demonstrated that DIA offers unbiased and 2–3-fold more comprehensive glycopeptide coverage, especially significantly fucosylated and combined fucosylated-sialylated glycans in individual patients, and 54 unique Hp glycopeptides exclusively present in HCC patients. We hope to extend this methodology for implementation for large scale glycoproteomics in the future.

## Methods

### Materials and Reagents

Iron(II) chloride tetrahydrate, iron(III) chloride, phosphate buffer saline (PBS), TTBS (TBS-Tween 20), formic acid (FA), sodium hydroxide (97%), (3-aminopropyl) trimethoxysilane (97%), triethylammonium bicarbonate (TEABC), bis(*n*-succinimidyl)-substrate (DSS), and hemoglobin were obtained from Sigma-Aldrich (St. Louis, MI, USA). ZIC-HILIC columns (5 μm,100 Å, 150 × 4.6 mm), acetonitrile, and C18 zip-tips were purchased from Merck-Millipore (Darmstadt, Germany). Tetraethyl orthosilicate (≥98%), phenyl ether (99%), and oleylamine (80–90%) were purchased from Acros Organics (Fair Lawn, NJ, USA). Trifluoroacetic acid (TFA) and ethyl acetate were purchased from the Wako pure chemical industry (Osaka, Japan). Dithiothreitol (DTT) and iodoacetamide (IAM) were purchased from JT Baker (Phillipsburg, NJ, USA). Modified sequencing-grade trypsin was purchased from Promega (Madison, WI, USA).

### Serum Samples

Participant recruitment and serum sample collection were approved and followed by the Institutional Review Board on Biomedical Science Research, Academia Sinica, and National Taiwan University Hospital (NTUH). Informed consent was obtained from all participants in this study which include HCC (*n* = 5) cases and HBV (*n* = 5) cases. The clinical features of patients with HCC and cirrhosis are summarized in Table S1. Samples were aliquoted and stored at −80 °C until further use.

### Preparation of Hemoglobin Conjugated Magnetic Nanoparticles (Hb@MNPs)

The synthesis of magnetic nanoparticles (Fe_3_O_4_) was obtained by the coprecipitation of FeCl_2_ and FeCl following previous protocols.^35^ Amine surface coating was achieved by a sol–gel process using tetraethyl orthosilicate (TEOS), followed by the addition of 3-aminopropyltri-methoxysilane (APS). The bifunctional linker, suberic acid bis-*N*-hydroxysuccinimide ester (DSS), was then used as the cross-linker with aminosilane MNPs with hemoglobin (Hb) to obtain the Hb@MNPs. A methoxy ethylene glycol (MEG) with a terminal amino functionality was reacted with the terminal *N*-hydroxysuccini-mide linkers on the MNPs to block nonspecific binding to the unreacted linker. After magnetic separation, the MNPs were washed with phosphate-buffered saline (PBS) three times.

### Hb@MNPs Selectivity Capture Haptoglobin

For each patient, 20 μL of serum was diluted in 60 μL of PBS buffer, and 70 μg of Hb@MNPs was then added to the mixture and incubated at room temperature for 1 h. After collecting the attached haptoglobin on Hb@MNPs by a magnet, the supernatant was removed and washed with 500 μL of TTBS buffer twice followed by sequential washing with ddH_2_O. The purified haptoglobin was eluted from the nanoparticles with 100 μL of elution buffer (1/49/50, TFA/H_2_O/ACN, v/v/v) in a vortex shaker at room temperature for 30 min. After purification, all samples were subjected to double digestion with sequencing grade Glu-C and trypsin.

### ZIC-HILIC Stage-Tips for Glycopeptide Enrichment

The ZIC-HILIC Stage-Tips were obtained by adapting a previously reported procedure with some adjustment.^36,37^ The ZIC-HILIC Stage-Tips were prepared by capping at one end with a 20 μm polypropylene frits disk (Agilent) enclosed in a tip-end fitting, loaded with 10 mg of ZIC-HILIC resuspended in deionized water (50 μL), loaded into a 200 μL tip, and centrifuged at 3000 rpm for 2 min, twice. Then, the surface was flattened by adding 50 μL of deionized water and centrifuging at 6000 rpm for 4 min. Prior to use, the Stage-Tips were preconditioned with 50 μL of 80%ACN/0.5% TFA (v/v) and then centrifuged at 3000 rpm for 10 min, twice. The digested serum samples were resuspended in 80% ACN/0.5% TFA (20 μL), loaded into the Stage-Tips, and centrifuged at 3000 rpm, for 10 min; then, the flow-through was reloaded into Stage-Tips and centrifuged at 4000 rpm for 8 min. The bound glycopeptides were washed with 20 μL of 80% ACN+0.5% TFA twice. The glycopeptides were then eluted three times with 20 μL of 0.5% FA, followed by centrifugation at 3000 rpm for 10 min, another 4000 rpm for 8 min, and another 5000 rpm for 6 min, then desalted with C18 zip-tip, and dried. Lastly, the glycopeptides were resuspended with 0.1% FA and spiked with iRT for LC-MS/MS analysis.

### LC-MS/MS Analysis

Mass spectrometry analysis was performed on an Orbitrap Fusion Tribrid mass spectrometer connected to an UltiMate 3000 RSLCnano System (ThermoFisher Scientific, Bremen, Germany) equipped with a nanospray interface (Proxeon, Odense, Denmark). All enriched glycopeptides spiked with iRT peptides were separated by the nanoflow UHPLC system using a capillary C18 column (Waters, nanoEase, 130 Å, 1.7 μm, 75 μm × 500 mm) and separated with a segmented 90 min gradient with the mobile phase (Buffer A, 0.1% FA in water; Buffer B, 100 ACN with 0.1% FA) with 2–85% buffer B at 250 nL/min flow rate. Tandem MS was performed by fixed higher-energy collision dissociation (HCD) for the DIA method and HCD product-dependent stepped HCD with normalized collisional energy (NCE) of 27%, 35%, and 43% for DDA workflow. The DIA data sets were acquired using the following parameters: For master scan range of 400–2000 *m*/*z*, MS resolution of 120,000, standard AGC target, and maximum injection time of 50 ms. For MS2, the MS/MS scan was performed in HCD mode with the following parameters: using 16 Da isolation window over 800–1600 *m*/*z* precursor and scan range of 110–2000 *m*/*z*, resolution 30,000 with dynamic maximum injection time, normalized AGC target of 400, and normalized collision energy of 31%. All data were acquired in positive polarity and profile mode. As for the DDA data sets, details are provided in the Supporting Information.

### Data Processing

Raw data were converted to .mgf files. The .mgf files were used to count the number of MS/MS spectra containing any two of the three common diagnostic oxonium ions (*m*/*z* 366.11 for HexHexNAc+, 204.08 for HexNAc+, and 138.06 for HexNAc+ fragments) with S/N ≥ 10 as derived from glycopeptides. Three other diagnostic oxonium ions of sialylation (*m*/*z* 274.09 for Neu5Ac-H2O+, 292.09 for Neu5Ac+, and 657.24 for Neu5Ac-Hex-HexNAc+) were also used as evidence of MS/MS spectra derived from sialylated glycopeptides.

For the DDA data set, Byonic (v3.6, Protein Metrics) and Proteome Discoverer 2.5 were used for identification and quantitation of intact glycopeptides. The raw data were queried for Glu-C and tryptic peptides with a maximum of two mis-cleavage sites, a precursor ion mass tolerance of 10 ppm, and a fragment ion tolerance of 0.05 Da for HCD spectra. Protein FASTA files, including Human (Swiss-Prot database, v2021-05-06, total 20,324 sequences from human), were used for protein identification. 164 built-in human N-glycans were integrated from a Byonic default database, including 15 human IgM 57 human plasma N-glycans, and 182 human N-glycans no multiple fucose without sodium adduct were used for identification of glycan composition. Methylthio (C) was selected as fixed modification; deamidation (NQ) and oxidation (M) were selected as variable (common) modifications. N-Glycans were selected as “rare”. In the main text, glycans were described as NxHxFxSx: Nx, number(x) of *N*-acetylglucosamoine (GlcNAc); Hx, number (x) of linked mannose or galactose on antenna; Fx, number (x) of fucose; Sx, number (x) of sialic acids. For example, N4H5F1S2 represents the monofucose-bisialyl-biantennary glycan. The maximum of total common modification was set as 4, and rare modification was set as 1. The score of identified glycopeptides was higher than 30, and confident identification of glycopeptides was set at >100; reversed peptide sequence identification was also considered with a protein FDR of 1% or 20 reverse counts.

For the DIA data set, Byonic (v3.6, Protein Metrics) and Skyline software were used for identification and quantitation of intact glycopeptide. The DIA raw data was first processed using Byonic with the same parameters as DDA data analysis. The DIA .mzID files derived from Byonic were used to construct the spectral library using Skyline software. Based on their unique monoisotopic mass (Da), the glycan database was submitted manually to the peptide modification setting in Skyline. The iRT calibration curve was also confirmed “successful” with *R*^2^ > 0.995. A 10 ppm of mass tolerance was used for peak extraction. The label-free quantitation was further processed by using chromatographic alignment with *m*/*z* and retention time of identified intact glycopeptide; the precursor similarity or isotope Dot product (idotp) and library score match (*q*-value < 0.05) were also considered. The final list of glycopeptides was then modified manually to ensure the true positive identifications based on their peptide b/y ions, its unique glycan fragments, and oxonium ions transitions.

### Data Analysis

The quantitation of intact glycopeptides from serum Hp proteins were exported from Skyline by using DIA analysis. Glycopeptide with precursor and at least 5 fragment signals including, peptide b/y ions, its unique glycopeptide fragments (Y1), and oxonium ions (common glycopeptides with *m*/*z* 366.11 for HexHexNAc+, 204.08 for HexNAc+, and 138.06 for HexNAc+; sialo-glycopeptides with 274.09 for Neu5Ac-H2O+, 292.09 for Neu5Ac+, and 657.24 for Neu5Ac-Hex-HexNAc+), were selected for further quantitation. The glycopeptide’s relative abundance was obtained by peak areas and normalized to the most abundant glycopeptide per patient.^38^ The fold change of glycopeptide abundance was analyzed by the student *t* test and considered significant with a *p* value < 0.05. All tests were two-tailed, and *p* values < 0.05 were considered significant. These differentially expressed glycopeptides were further analyzed by hierarchical clustering.

## Results and Discussion

### Design of Spectral Library-Assisted DIA-MS Workflow

**Figure 1:**
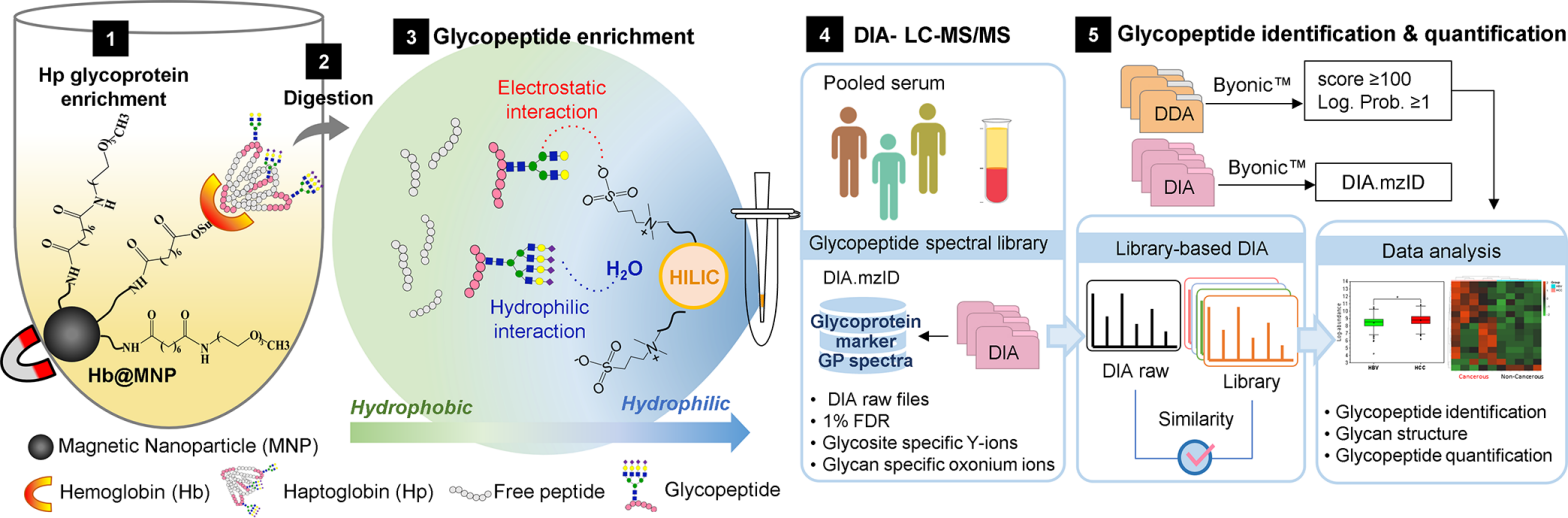
Schematic diagram of the integrated platform for identification and quantitation of haptoglobin glycopeptide using DIA-MS. By combining hemoglobin-conjugated magnetic nanoparticles (Hb@MNPs) specific to haptoglobin with a well-established ZIC-HILIC separation strategy, we expect highly sensitive and specific haptoglobin glycopeptide enrichment. We established an Hp-specific glycopeptide spectral library using DIA data from pooled serum samples, integrating Byonic and Skyline software. Individual patient Hp was analyzed via single-shot DIA spiked with iRT for N-glycopeptide identification and label-free quantitation in the HCC and HBV groups. Statistical analysis evaluated differentially expressed site-specific N-glycopeptides between these cohorts.

Using Hp as a model glycoprotein biomarker, we developed an integrated platform for identification and quantitation of Hp glycopeptide using the spectral library-assisted DIA-MS method (Figure 1).

In this study, quantitative comparison of glycosylation patterns will be performed in serum from HCC (*n* = 5) and HBV (*n* = 5) patients. We have demonstrated that functionalized magnetic nanoparticles (MNP) demonstrated excellent enrichment performance in sensitivity and specificity to facilitate targeted protein detection.^39−42^ Thus, the first step will involve design and fabrication of affinity MNPs (Figure S1) to provide specific purification of Hp protein from serum. Hemoglobin (Hb), which possesses high affinity with Hp,^19,43,44^ was conjugated on magnetic nanoparticles (Hb@MNPs) to purify Hp from patients’ sera, which were optimized to show good purity and recovery validated by SDS-PAGE (Figure S2) with high recovery (Step 1) (Table S2). Following digestion of purified Hp protein (Step 2), Hp glycopeptides were then enriched using the ZIC-HILIC Stage-Tips strategy (Step 3).^37^ Based on the hydrophilic glycopeptides partitioned in the hydration layer surrounding the stationary phase and the less-polar free peptides in the mobile phase, we expected that this strategy was able to improve the glycopeptide enrichment performance. To improve the glycopeptide coverage of HP, a Hp-specific glycopeptide spectral library was established by the DIA data set from pooled serum samples using the integration of Byonic and Skyline software (Steps 4–5). The Hp from individual patients was analyzed by single-shot DIA spiked with iRT for identification and label-free quantitation of N-glycopeptides in the HCC and HBV groups. Finally, statistical analysis was performed to evaluate the differentially expressed site-specific N-glycopeptide in HCC and HBV groups.

### Establishing Spectral Library to Enhance Glycopeptide Identification Coverage

Though direct data deconvolution of DIA data is commonly applied for label-free quantitation, high coverage and quality reference spectral libraries can facilitate targeted peptide signal extraction to enhance identification coverage.^38^ Because the different data acquisition modes in DDA and DIA will cause different precursors and fragmentation patterns, the spectral libraries can be constructed by DDA, DIA, or hybrid DDA/DIA data sets that may have different effects to improve the glycopeptide identification coverage. Prior to establishing the spectral library, standard Hp protein was used as a model to test the workflow and determine the optimum DIA window size (Da) and HCD fragmentation energy (Figure S3). Comparing the different scanning windows (8, 12, 16, and 20 Da), 16 Da window size outperforms the other window sizes by 14–25% more glycopeptides (Figure S3A). The HCD fragmentation energy (27, 31, 35, 39, and 49) also affects the identification coverage (Figure S3B). The combination of 16 Da window size and HCD 31 collision energy have the highest glycopeptide identification for a total of 209 glycopeptides.

**Figure 2:**
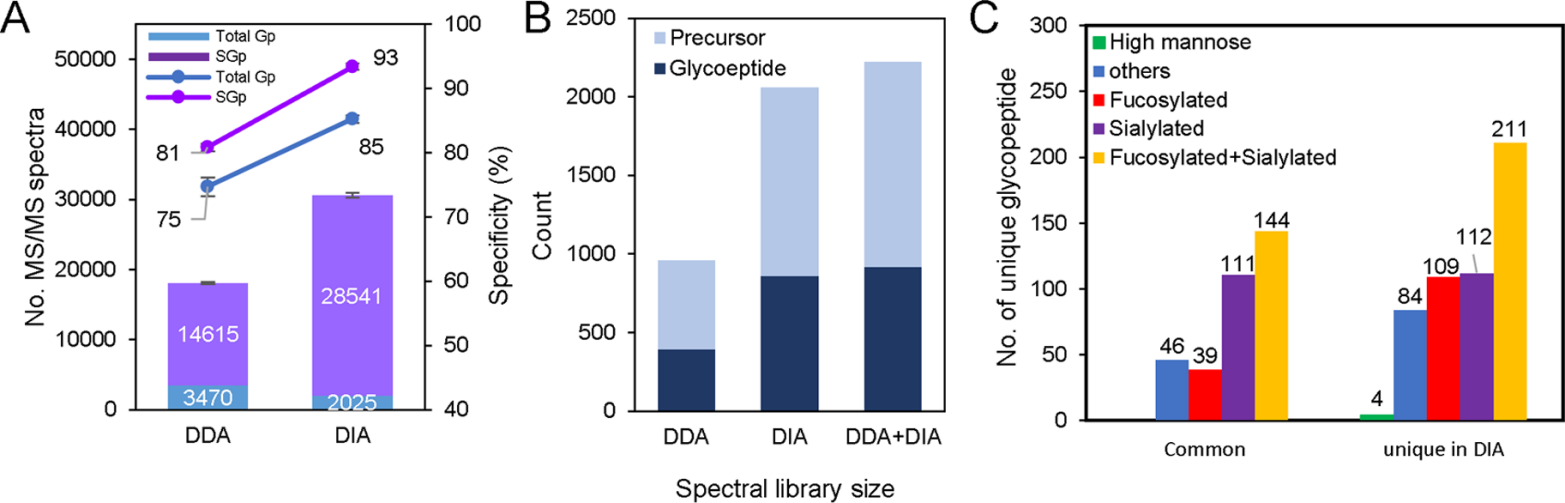
Construction and comparative analysis of spectral libraries generated using DDA and DIA data sets obtained from triplicate analysis of the HCC serum sample. (A) Comparison of the number of MS/MS spectrum containing oxonium ions from glycopeptide (GP) and sialylated glycopeptide (SG) between DDA and DIA methods. (B) Total number of precursors and glycopeptides included in the 3 spectral libraries constructed by DDA, DIA, or hybrid DDA/DIA data sets. (C) Glycan type distribution from glycopeptides commonly present in the DDA and DIA libraries or unique glycopeptides in the DIA spectral library.

Based on the above optimized DIA strategy, next, we generated the DDA and DIA data sets to construct the glycopeptide spectral libraries from HCC serum samples. To ensure the sufficient coverage of glycosylation pattern, the data sets for library construction were generated by using triplicate analysis of one HCC serum sample. The glycopeptides were enriched from pooled HCC serum using the ZIC-HILIC strategy in triplicate, followed by DDA and DIA LC-MS/MS analysis. We first evaluate the performance of glycopeptide enrichment at the MS/MS and glycopeptide levels. To evaluate the glycopeptide coverage bypassing their identification challenge, the glycopeptide enrichment specificity was determined by counting the numbers of glyco-oxonium ion-containing MS/MS spectra among the total number of MS/MS spectra. For sialylated glycopeptides, similarly, the specificity was calculated by number of MS/MS spectra containing sialylated oxonium ions, relative to the number of glycopeptide spectra. We count the glycopeptide MS/MS by the presence of diagnostic glyco-oxonium ions of HexHexNAc+ (*m*/*z* 366.11), HexNAc+(*m*/*z* 204.08), and HexNAc+ (*m*/*z* 138.06) in the mass spectra. As shown in Figure 2A, the DIA mode obtained significantly higher glycopeptide MS/MS counts (>30,000 MS/MS spectra) and good enrichment specificity (85.1%) than the results from the DDA (>17,000 MS/MS spectra and 75% enrichment specificity) method. Among the glycan types, our ZIC-HILIC stage-tip shows dramatic preference to enrich sialylated glycopeptide, evidenced by the oxonium ions of Neu5Ac-H2O+ (*m*/*z* 274.09), Neu5Ac+ (*m*/*z* 292.09), and Neu5Ac-Hex-HexNAc+ (*m*/*z* 657.24). Among all the spectra, furthermore, DIA shows better detectability to identify a very high percentage (>28,000 MS/MS spectra and 93% enrichment specificity) of sialylated glycopeptide MS/MS spectra compared to the DDA method (>14,000 MS/MS spectra and 80% enrichment specificity) (Figure 2A).

For construction of spectral libraries, the triplicate DDA and DIA data sets were then processed by Byonic (1% FDR) and Skyline software (cutoff *q*-value < 0.05) for glycopeptide identification. The glycopeptide spectra from each library were manually filtered to include corresponding oxonium ions and glycan fragment features. Taking advantage of different data generation for precursors and fragmentation patterns in DIA and DDA, where DDA provides in-depth fragmentation information for peptide identification while DIA offers high reproducibility and sensitivity for quantification,^32^ a hybrid library of glycopeptide mass spectra was constructed by combining the triplicate DDA and DIA data sets which were obtained by the same procedure as described earlier. Figure 2B shows the comparison of glycopeptide identification results using spectral library sizes constructed from DDA, DIA, and hybrid DDA/DIA data sets. Compared to the conventional DDA library (394 glycopeptides from 961 precursors), DIA and hybrid DDA/DIA libraries offer dramatically enhanced coverage of 860 glycopeptides from 2059 precursors and 914 glycopeptides from 2223 precursors of Hp glycopeptides, respectively. Among the total of 914 glycopeptides from the combined data set, only 340 glycopeptides (38%) were common from the three library-based DIA data sets, while the DIA library contained more than 50% of unique glycopeptides from the DIA data set (502 glycopeptides, 56%) than the DDA data set (54 glycopeptides, 6%) (Figure S4). Furthermore, we analyze the glycan type of the common and the unique glycopeptides generated from DDA and DIA data sets (Figure 2C). Based on the ZIC-HILIC Stage-Tip enrichment, the DIA method detected 46.5% more unique combined fucosylated-sialylated glycopeptides than commonly shared glycopeptides. Most dramatically, a significant increase (179.5%) in uniquely identified fucosylated glycopeptides was also observed in the DIA data set. Taken together, these results indicate that the DIA method provides better detectability for glycopeptides, especially the combined fucosylated-sialylated glycopeptides, thus leading to higher coverage for total glycopeptides and sialylated glycopeptides than the DDA method.

### Comparison of MS/MS Spectral Quality in DDA and DIA

**Figure 3:**
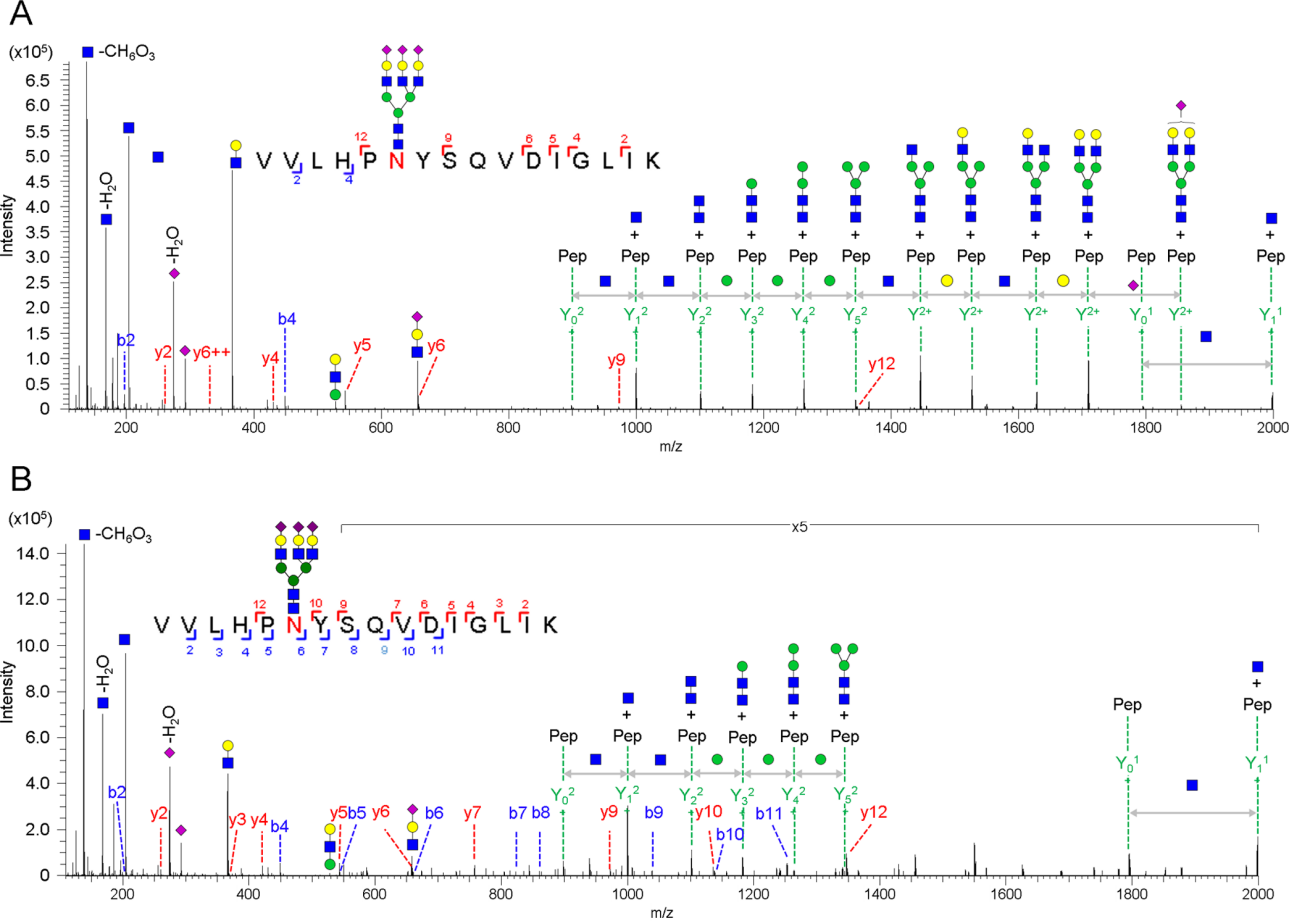
Representative MS/MS spectra of identified Hp glycopeptide from HCC serum. Comparison of 2 identical trisialyl-triantennary glycans (A) from DDA (*m*/*z* 1552.34, *z* = 3+, Byonic score = 673.4) and (B) from DIA (*m*/*z* = 1553.0088, *z* = 3+, Byonic score = 939.5). Blue squares, GlcNAc; green circles, Man; yellow circles, Gal; purple diamond, sialic acid.

In order to evaluate the quality of MS/MS spectra for incorporation into the spectral library, we manually validate the MS/MS spectra from DDA and DIA raw data sets using Xcalibur and Byonic software. Figure 3 shows two representative MS/MS spectra of identical N-glycopeptide VVLHPN^241^ YSQVDIGLIK with complex type of trisialyl-triantennary glycan from DDA and DIA raw data, respectively. The DDA spectrum possesses a typical glycopeptide pattern of strong signals of oxonium ions and glycan fragments with very low intensities of peptide-derived b/y ions (b2, b4, y2, y4, y5, y6, y9, y12) (Figure 3A, Byonic score of 673.4).

Though the deconvoluted MS/MS spectra of the same glycopeptide by direct DIA has lower intensity of glycan fragments (Figure 3B), the spectra recover more b/y ions (b2-b11, y2-y7, y9, y10, y12) compared to DDA spectra and offer unambiguous identification with a higher Byonic score (939.5). Both DDA and DIA MS/MS spectra show the expected dominant oxonium ion pattern (*m*/*z* 366.14 for HexHexNAc+, 204.09 for HexNAc+, 138.06 for HexNAc-CH_6_O_3_, 168.07 for HexNac-_2_H_2_O, 274.09 for Neu5Ac-H2O+, and 292.09 for Neu5Ac+) and the aforementioned b/y ions and intense core glycan fragments, including Y0 (naked peptide), Y1 (peptide+HexNAc), Y2 (peptide+2HexNAc), Y3 (peptide+2HexNAcHex), Y4 (peptide+2HexNAc2Hex), and Y5 (peptide+2HexNAc3Hex), which provided good spectra similarity to the fragmentation pattern in the library for confident annotation for both the glycan and peptide sequence. In addition, manual inspection was conducted to ensure the validity of our data. Figure S5 compares the fragment similarity between representative glycopeptides obtained with Byonic scores of different ranges: (A) <100, (B) 100–200, and (C) >300 from DDA and DIA data sets. As shown in the figure, there is a high degree of similarity in the fragments between the DDA and DIA data sets, demonstrated by a positive correlation (*r* ≥ 0.9) across the entire score range. Furthermore, score distribution and matched peaks of the DIA result from an HCC serum sample are also summarized in Figure S6. The largest group (72.2%) of the spectra have Byonic scores higher than 200 (Figure S6A). To keep stringent identification, it is important to note that we exclude the relatively less confident spectra with <100 score (12.7%). Figure S6B shows the score distribution of different glycan types in the same data set. These results indicate that both methods provide consistent and reliable results for glycan structure analysis.

We also examined the DIA MS2 spectra from different score ranges in Figure S7. Figure S7A presents the spectra identified with Byonic score less than 100. In this category, most of the peaks matched by Byonic software were glycan fragments, and only few peaks were matched with peptide fragments plus partial oligosaccharide. For the spectra with scores from 100 to 150 (Figure S7B), the number of matched peptide-related fragments (such as Y ions or b,y ions plus a HexNAc) was increased. For the score range of 150–200 (Figure S7C) and >200 (Figure S7D), good-quality spectra containing mostly glyco-oxonium and Y-type ions and a few peptide-related fragments were observed. These spectra reveal that the score threshold of 100 already provided sufficient accuracy for glycopeptide identification in a single protein, which indicates sufficient fragmentation ions and confident glycopeptide identification by the library-based DIA method. Previous studies have also suggested the criteria of a Byonic score ≥100 to ensure confident identification, while good quality GPSMs showed a score ≥300.^45^

### Evaluation of Library-Based DIA Performance

**Figure 4:**
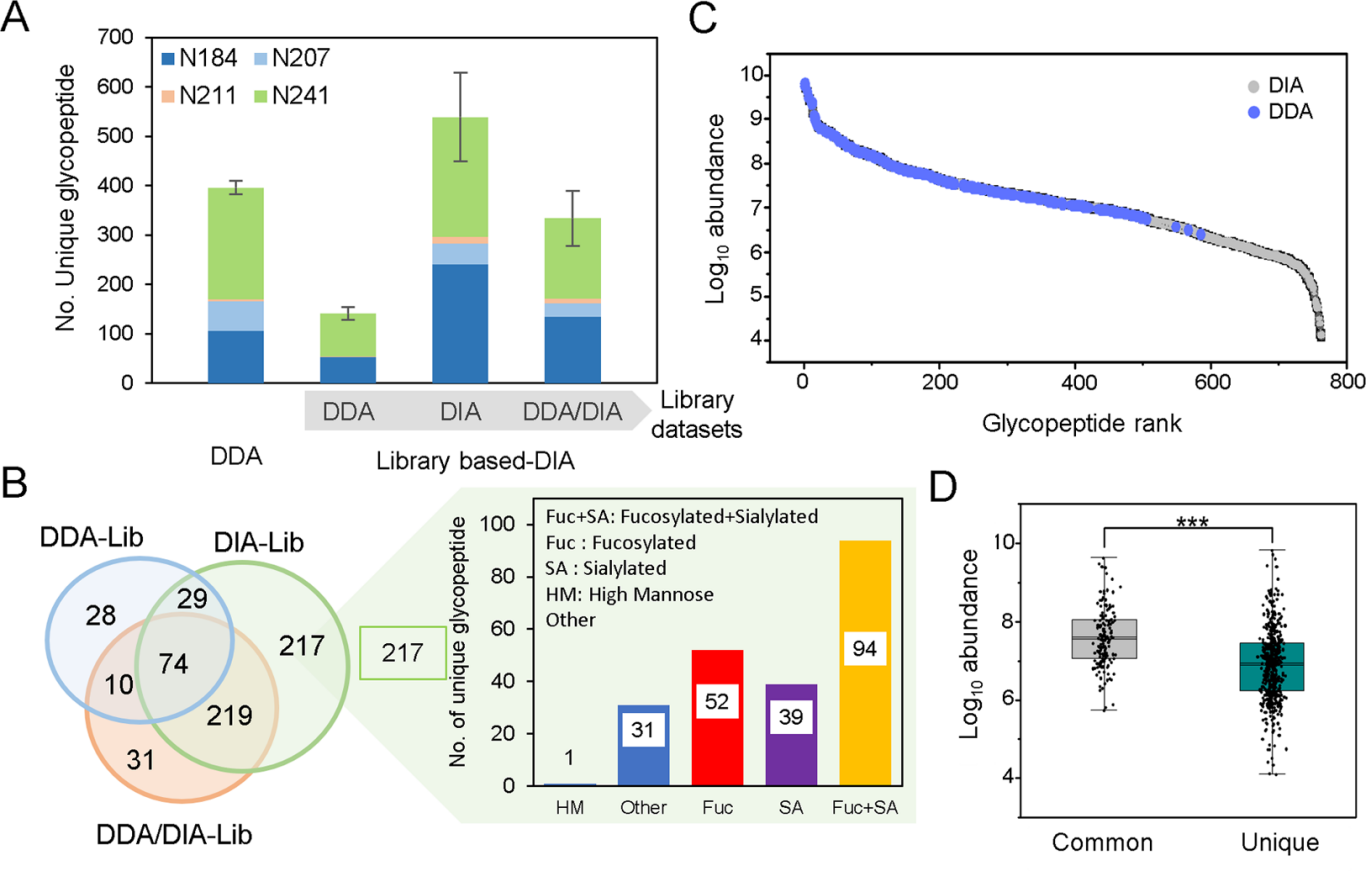
Comparison of single-shot DDA and DIA analysis using 3 technical replicates of the HCC serum sample. (A) Comparison of identified Hp glycopeptide from each glycosite between the DDA and library-based DIA methods. (B) Venn diagram of overlapping glycopeptides identified from the DDA, DIA, and hybrid DDA/DIA libraries. The glycan type distribution from the uniquely identified glycopeptide from the DIA library (217 glycopeptides) was shown to demonstrate the predominant identification of complex types of glycan from library-based DIA. (C) The abundance distribution of all glycopeptides and (D) comparison of common and unique glycopeptide abundance between the DDA and DIA methods.

With the established spectral libraries, we next investigated the glycopeptide identification performance using library-based single shot DIA and compared the impact of different spectral sizes to the identification coverage. Specifically, a single shot DIA data set was generated by triplicate analysis of one HCC serum sample and the raw data were processed against the 3 above-mentioned spectral libraries, constructed from the DDA data set (size of 394 glycopeptides), DIA data set (size of 860 glycopeptides) and hybrid DDA/DIA data set (size of 914 glycopeptides). A full list of glycopeptide spectral library generated from DDA, DIA, and hybrid DDA/DIA data sets can be found in Table S3. The glycopeptide identification for DDA was performed by using Byonic software and filtered with high confidence glycopeptide (1% FDR, Byonic score ≥ 100, and Log probability ≥ 1). Among the four N-glycsosites in Hp, the number of identified glycopeptides across different sites have similar patterns by either DDA or DIA methods (Figure 4A). In terms of number of glycopeptides, the identification results show that single shot DIA mapping to DIA library has the best coverage (539 glycopeptides), which significantly outperformed the coverage using the single shot DDA method (396 glycopeptides) (Figure 4A). A full list of the identified glycopeptide can be found in Table S4. The data types to construct the spectral library have profound effects in the data deconvolution. Interestingly, though the hybrid DDA/DIA library has a similar size of glycopeptide spectra compared to the DIA library, it shows lower recovery in glycopeptide identification (334 glycopeptides) (Figure 4A), which is likely due to the different fragmentation pattern in the DDA and DIA modes for lower spectral similarity.

Siyal et al. previously reported that, while extensive DIA libraries cover more peptides or proteins, they do not always yield the best results. This is due to the large number of spectra increasing false positives and the potential mismatch in fragmentation patterns between varied sample sizes, affecting sensitivity.^33^ Thus, the library size and spectral similarity (fragmentation ion pattern) play crucial roles in mapping the low abundant glycopeptides. As the size of the library increases, it may cause the likelihood of different fragmentation patterns and higher search space, leading to lower identification results.

To further study the effects of libraries on their corresponding glycopeptide identification, we examined the common and unique features of the 141, 539, and 334 identified glycopeptides using the three libraries from DDA, DIA, and hybrid DDA/DIA data sets, respectively (Figure 4B). The overlapping of the three results show that a large portion (35%, 217 glycopeptide) was uniquely identified from the DIA library, while only 28 and 31 glycopeptides were uniquely identified from the DDA and hybrid DDA/DIA libraries, respectively. Furthermore, we analyze the glycan type distribution from glycopeptides uniquely identified from library-based DIA (Figure 4B). The glycan types are defined into four categories, including fucosylation, sialylation, comodification with fucosylation and sialylation, and “other” glycans. In this study, the other glycans refer to those which have simpler glycan structures and do not contain fucosylated, sialylated glycans, or high mannose-type glycans. Among the 217 glycopeptides exclusively identified from the DIA library, it is noted that the complex type of combined fucosylated-sialylated glycopeptides (*n* = 94) represents the largest group, compared to 52 fucosylated, 39 sialylated, and 32 other glycopeptides (Table S5). Recent studies have reported that the increase in fucosylation and sialylation has significant association to liver diseases, especially HCC. The observed superior detectability of combined fucosylated-sialylated glycans suggests its better suitability to analyze the glycan type distribution for understanding the functional implications of glycans, identifying disease biomarkers, and unraveling biological processes related to glycosylation.^17,22,39,46^ In addition, we also compare the glycopeptide abundance distribution between the DDA and DIA methods. As shown in the glycopeptide abundance index (Figure 4C), low abundance glycopeptides were mostly identified by the DIA method. Furthermore, the commonly shared glycopeptides between the DDA and DIA methods have relatively higher abundance distribution compared to the lower abundance of unique glycopeptides (Figure 4D). The distribution for most unique glycopeptides in the low abundance range additionally confirmed the ability of library-based DIA to recover low abundant glycopeptides. In summary, these results indicate that DIA provides more information from low abundance glycopeptides which are missing in the DDA method.

### Personalized Hp Glycoproteomic Profiles in HBV and HCC Patients

Encouraged by the above results, we further construct a glycopeptide spectral library generated from DIA data sets of all patient sera (1208 glycopeptides) (Table S6) and applied the spectral library-assisted single shot DIA strategy to establish the personalized Hp glycoproteomic profiles of individual patients with HCC (*n* = 5) and HBV (*n* = 5). According to the World Health Organization (WHO), the incidence of HCC cases is higher in males than females (male-to-female ratio: 2.66:1).^47^ Within a limited case number in our study, our study employed a gender distribution of HCC samples with dominant male samples, while we maintained a ratio of 2:3 for HBV samples. A larger sample size with appropriate male-to-female ratio would be beneficial for future research to ensure broader applicability and to address potential biases. For each patient, 20 μL of serum was used for analysis. Based on BCA protein assay results, 20 μL of serum is about 1.4 mg of total serum protein. We also performed ELISA analysis for the Hp concentration in each patient sample. On average, 20 μL of serum roughly consists of 5 μg of Hp protein enriched under the condition of the 2:7 serum:Hb@MNPs ratio (v/v). Because the serum proteins from different patients may have more complex and heterogeneous glycosylation patterns, DDA analysis for individual patients was also performed as a control to compare the identification performance and glycan type distribution profile of individual patients. The glycopeptide identification was confidently achieved with stringent criteria using Byonic (Score ≥ 100, log Probability ≥ 1)^45,48,49^ and Skyline software for DDA and DIA, respectively.

**Figure 5:**
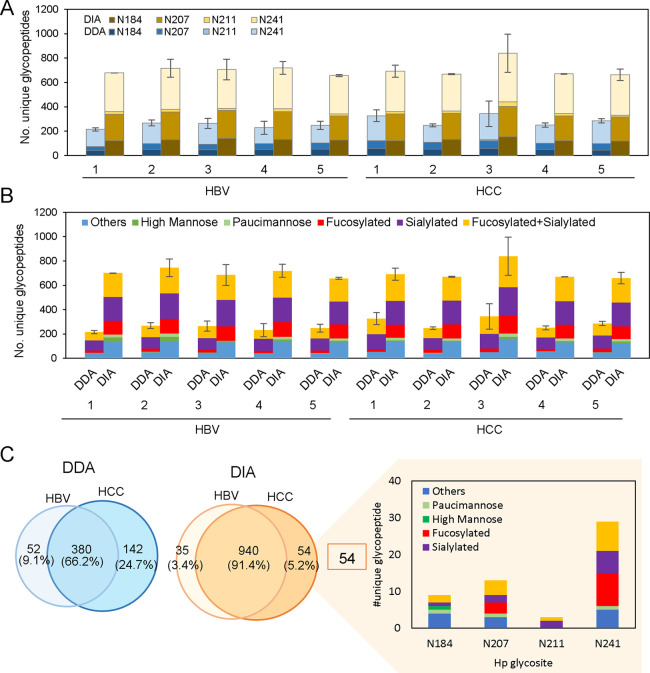
Comparison of DDA and DIA identification result for individual patient sera. Identification of unique Hp glycopeptides (A) per N-glycosylation site and (B) per glycan type distribution from individual HCC (*n* = 5) and HBV (*n* = 5) patients. (C) Venn diagram of overlapped glycopeptide from DDA and DIA across each disease group. The glycan type distribution from 54 glycopeptides identified in the DIA method.

Figure 5 shows the comparison of site-specific glycopeptide identification results across the HBV and HCC patients using DDA and DIA.

Compared to the five HBV patients with profiles of 432 unique glycopeptides and average of 245.2 ± 22.5 glycopeptides, the DDA analysis results show that a slightly higher number of 522 unique glycopeptides and average of 291 ± 43.5 glycopeptides were identified from the HCC (*n* = 5) patients, in which most glycopeptides were distributed at the N-241 site (Figure 5A). Using the DIA approach mapping to the DIA library, nearly 2-fold higher coverage was observed for both HBV and HCC patients, achieving a total of 975 and 994 unique glycopeptides identified from HBV and HCC patients, respectively. Among the 4 glycosylation sites, it is noted that most of the glycopeptides were distributed at N-241 (41.2%) and N-207 (31.2%) sites (Figure 5A). Combining all data sets from HBV and HCC patients, a total of 1246 glycopeptides were identified. Among them, only 357 (28.6%) glycopeptides were commonly presented in the two patient cohorts, suggesting the different glycosylation profile of Hp between HCC and HBV as well as heterogeneity among the individual patients. Comparing the methodology, it is important to note that DIA contributed to significantly more uniquely identified glycopeptides 672 (54%), compared to 217 (17.4%) glycopeptides uniquely presented in DDA (Figure S6). These results indicate that the library-based DIA method achieved superior performance in identification coverage (∼2–3-fold) compared to the DDA method, which helps for better resolving the heterogeneous glycosylation profile among individual HCC and HBV patients.

The aberrant glycosylations, such as fucosylation and sialylation, have been reported to be closely associated with cancer progression as promising biomarkers.^22,23,50−52^ Thus, we further analyze the glycan type distribution of the 4 glycosylation sites across individual HCC and HBV patients (Figure 5B). Based on the enrichment by ZIC-HILIC Stage-Tips, Hp shows the presence of the highest proportion of sialylated glycopeptides (37.8% and 31.4%) and combined fucosylated-sialylated (35.6% and 31.5%) glycopeptides, followed by fucosylated glycopeptides (8.7% and 15.3%) from DDA and DIA analysis, respectively. Interestingly, DIA shows different degrees of enhancements on the glycan types across all serum samples. Compared to the DDA results, DIA shows a dramatic increase on the detection of high mannose (12.2-fold) and fucosylated (5.4-fold) glycopeptides, followed by combined fucosylated-sialylated (2.0-fold) and sialylated (2.3-fold) glycopeptides, respectively. A full list of glycopeptides identified in individual patient samples using DDA and DIA can be found in Table S7 and Table S8, respectively.

Comparing the overlapped glycopeptides between HBV and HCC groups in DDA and DIA (Figure 5C), the HCC group shows a higher number of unique glycopeptides than the HBV group in both DDA and DIA methods. From the total glycopeptide identification level, DIA (1029 glycopeptides, 130 glycans) outperforms DDA (574 glycopeptide, 141 glycans) across all patient serum samples. Importantly, the library-based DIA offers more common identification (91.4%, 940 glycopeptides) between HBV and HCC groups than the DDA method (66.2%, 380 glycopeptides). Such difference revealed the stochastic nature in DDA mode, especially for the negatively charged glycopeptides, and the advantage of more comprehensive coverage of DIA that may offer relatively unbiased comparative profiling for biomarker discovery. Thus, we further analyze the glycan type distribution of 54 glycopeptides that uniquely exist in HCC from the DIA method (Table S9). Compared to the methodological feature that sialylated glycopeptide is the top-ranking glycan in the DIA results (Figure 5B), interestingly, the DIA recovered much more unique fucosylated glycopeptides in the N-241 site of the HCC group, suggesting the dominance of fucosylation associated with HCC (Figure 5C).

Without further fractionation, our single shot DIA approach has achieved a comparable number of glycopeptides for individual patients compared to a recent study on targeted characterization of Hp from the pooled serum sample after applying depletion of abundant serum proteins and a high pH reversed-phase peptide fractionation strategy (8 fractions).^17^ In addition, compared to the reported haptoglobin glycopeptides (409 glycopepitdes) from HCC serum using the DDA method,^22^ our DDA (574 glycopeptide, 141 glycans) and DIA (1029 glycopeptides from 130 glycans) result provided 1.4- and 2.5-fold more glycopeptides across all patient samples, respectively. To the best of our knowledge, all the 54 unique Hp glycopeptides identified exclusively in HCC were not found in any previously reported HCC studies, indicating novel and HCC-associated glycosylation in haptoglobin using the DIA method.

### Basal and Alteration of Site-Specific Glycosylation of Hp in HBV and HCC

To explore the distinct patterns between individuals with HCC and those with HBV, we performed a quantitative comparison of the glycopeptide abundance between the HBV and HCC patients, determined by calculating peak areas through label-free quantitation using Skyline software. Statistical analysis was employed to analyze the differential glycopeptide signature as potential biomarkers to differentiate HCC and HBV.

With the large-scale glycoform data set of Hp, it is intriguing to explore the heterogeneous glycan type distribution. We first determined the top-ranking abundant N-glycopeptides in HCC at each glycosite and compared their abundance in HBV (Figure S9). The glycan abundance at N-184, N-207, N-211, and N-241 sites was normalized against the total sum of Hp glycopeptides at the corresponding glycosite. Average abundance and standard deviation were calculated across five individual patients within each group. The N-241 site has the largest number of glycopeptides. Interestingly, its top 10 most abundant glycoforms in HCC were dominated with sialylated glycans, with disialylated (N4H5S2) and monosialylated biantennary glycan (N4H5S1) as the most abundant glycoforms (Figure S9D). These two glycoforms are also dominant for N-184 and N-211 sites (Figure S9A,C), which is consistent with the Lubman group’s report using the DDA method in which N4H5S2 is the most abundant glycoform in HCC at N-184, N-207, and N-241 sites in HCC.^52^ Notably, most of these abundant glycoforms have similar abundance among HCC and HBV groups, indicating that distinguishing between HCC and the HBV group remains challenging when relying solely on the most abundant glycoform.

**Figure 6:**
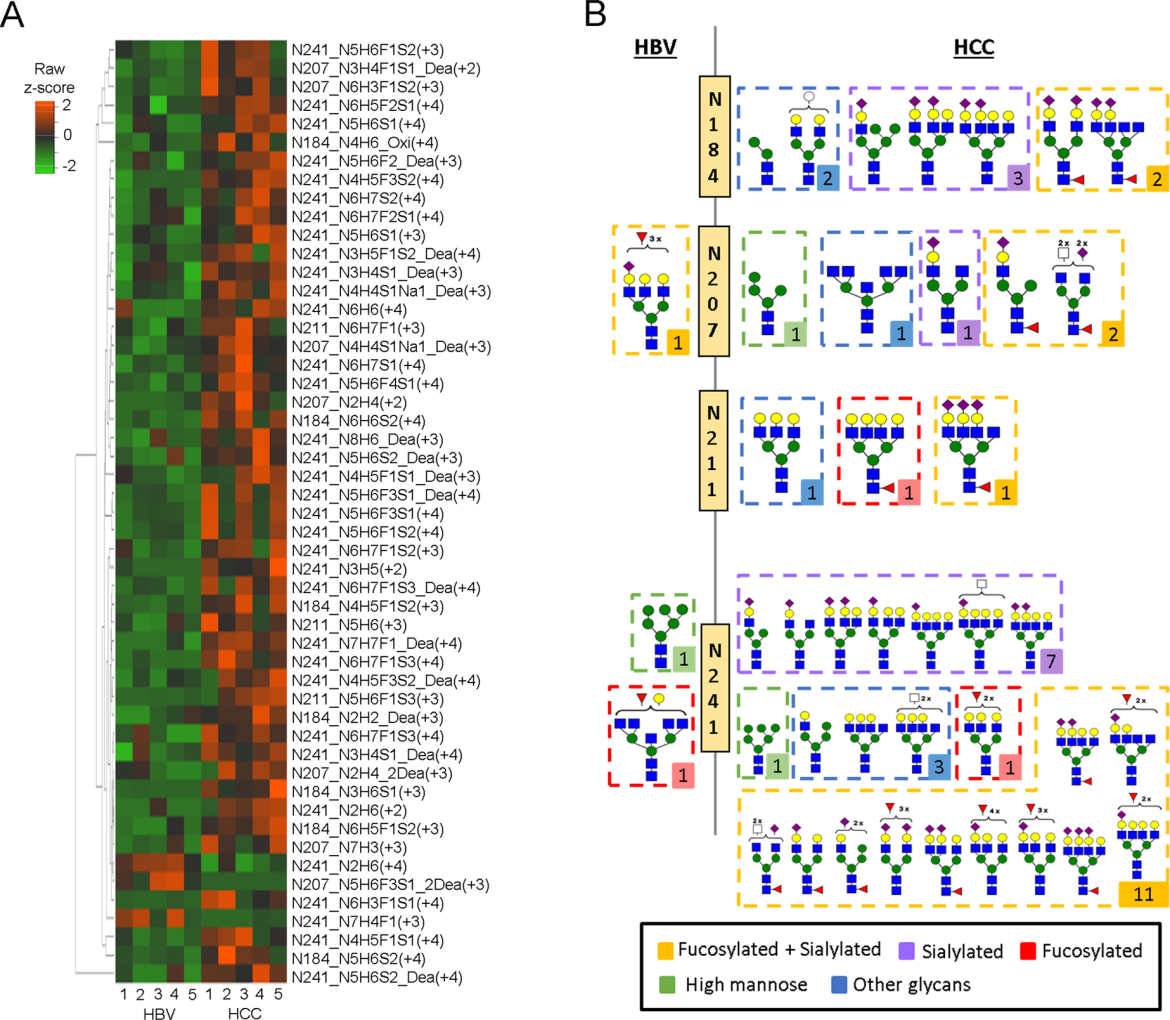
Quantification and differential abundance of glycopeptide identified from HCC and HBV patients using the library-based DIA method. (A) Heatmap of differential Hp glycopeptide precursor and corresponding glycans (shown by N-glycosite, glycan composition, peptide modification, and *m*/*z*) categorized by hierarchical clustering. Dea: Deamidation, Oxi: Oxidation. (B) Representative figure of glycan structures of glycopeptides enriched in either HCC group (*n* = 5) or HBV group (*n* = 5) in each Hp glycosite (N184, N207, N211, and N241). The hollow rectangle with the dashed line categorizes glycans into 5 groups (yellow: fucosyl and sialyl; purple: sialyl; red: fucosyl; green: high mannose; blue: other glycans). The numbers indicate the total counts of categorized glycan structures in each glycan type for the corresponding glycosite.

Finally, statistical analysis was employed to assess the fold change in glycopeptide abundance between HCC and HBV. The relative abundances of glycopeptides were normalized using min-max scaling. A total of 51 differentially expressed glycopeptide precursors, consisting of 4 high mannose, 13 sialylated, 4 fucosylated, 23 combined fucosylated-sialylated, and 7 other types of glycans (student *t*-test, *p* < 0.05), were subjected to hierarchical clustering analysis (Figure 6A). Their fold change and *p* value between HCC and HBV are also listed in Table S10. Generally speaking, most glycans with elevated abundance in HCC have complex structures of combined fucosylated-sialylated. Based on the heatmap clustering result, all the unique glycans per N-site that are differentially expressed in HBV and HCC are summarized in Figure 6B. Among the 23 combined fucosylated-sialylated glycopeptide precursors, 22 were significantly elevated and two glycopeptides have dramatically elevated HCC/HBV ratios, N5H6F1S3 (30.6 ± 11.3-fold) at N-211 site and N6H3F1S1 (29.1 ± 13.7-fold) at N-241 site, suggesting their potential as Hp marker for HCC detection. The result is in accordance with some published literature.^40−42^ For example, Zhu et al. reported that five combined fucosylated-sialylated N-glycopeptides at sites N-184 and N-241 were significantly elevated during the progression from nonalcoholic steatohepatitis (NASH) cirrhosis to HCC (*p* < 0.05).^22^ Most recently, Kohansal-Nodehi reported that significantly higher fucosylation, branching and sialylation of Hp glycans, and expression of high-mannose glycans were observed as the disease progressed from cirrhosis to early- and late-stage HCC.^23^ Interestingly, in our study, combined fucosylated-sialylated glycans present the dominant category in both HCC and HBV groups. However, a significant number of differentially expressed glycopeptides belonged to the HCC group; the result revealed that 45 and 3 glycopeptides were upregulated in HCC and HBV, respectively (Table S10). For example, the most dramatic difference occurs on a hybrid monosialylated triantennary (N3H6S1) glycopeptide on the N-184 site, with a significant none-to-all elevated level in the HCC group (2462.3 ± 766.8 HCC/HBV ratio, *p* value < 0.05), while a monosialylated-trifucosylated triantennary (N5H6F3S1) glycopeptide with double deamidated modification on the N-207 site was exclusively detected in the HBV group. In addition, our result also showed a number of tetra-antennary glycans in the HCC group (Figure 6B) out of 38 unique glycans; a total of 13 glycans (34%) were tetra-antennary glycans, which were elevated in the HCC group. It is important to note this result may diverge from other research groups that have identified tetra-antennary glycans as robust markers for distinguishing HCC from cirrhosis.^53,23^ This discrepancy might arise due to the different etiology in our sample; Zhu et al. discovered the impact on the Hp glycosylation profile in different etiologies of HCC patients. They discovered that tetra-antennary glycan was significantly expressed in both HBV and HCC related to HBV patients. Therefore, in agreement with our findings, the expression of tetra-antennary glycan in HCC patients was noteworthy but not sufficiently significant to be deemed as a reliable marker for HCC.^50^ In summary, these results revealed that combined fucosylated-sialylated glycans are predominantly present in HCC as a potential panel of biomarkers. The exclusive detection of the multiple sialylations and tetra-antennary glycoforms in all 5 HCC patients show promise to discriminate HCC from HBV; its potential utility will require further validation in more patients.

## Conclusions

In summary, by integrating magnetic nanoparticles and ZIC-HILIC Stage-Tips for highly specific enrichment of Hp-glycopeptide, we developed an integrated library-based DIA-MS platform that portrays the aberrations in site-specific N-glycopeptides of haptoglobin in patient serum among HBV and HCC disease groups. This study demonstrated the utility of high-quality spectral libraries to enhance single-shot DIA analysis for a deep glycosylation profile. Using the DIA data set-based library approach, we observed 2–3-fold more glycopeptide in the personalized Hp glycopeptide identification results, especially more complex types with high numbers of monosaccharides and multiple sialylations. This is likely due to the different fragmentation pattern between DDA and DIA modes for lower spectral similarity. Furthermore, the presence of more complex types and heterogeneity of glycans among patients may also cause lower detectability in the DDA mode, which can have higher recovery by spectral match using the DIA-library approach. Despite the advanced sensitivity, the described strategy has several limitations. The method’s success was dependent on high-quality spectral libraries; applications to other single proteins will require a sample-specific spectral library. To implement the current method for more complex samples at the glycoproteomic level, building a comprehensive glycoproteomic spectral library will require stringent validation on the quality of glycopeptide spectra; development of FDR control tools will be essential.

This integrated library-based DIA workflow offers high coverage label-free quantitation of site-specific N-glycosylation in serum protein biomarkers. Toward biomarker discovery, label-free quantitation revealed 3 and 48 unique glycopeptides significantly enriched from HBV and HCC, respectively. Combinations of sialylated and fucosylated tetra-antennary glycopeptides are significantly expressed in the HCC group, including N5H6F1S3 (30.6 ± 11.3-fold) at N-211, N6H3F1S1 (29.1 ± 13.7-fold) at N-241, and an exclusive presence of N3H6S1 (2462.3 ± 766.8) at N-184, suggesting their potential as Hp markers for HCC detection. We expect that our strategy would be an initial catalyst for improving deep glycoproteome coverage. Moreover, the current findings may inspire further investigation on larger cohorts from healthy controls and other liver disease groups; our strategy may offer a high-performance alternative on glyco-biomarker screening for HCC or other associated diseases. For further validation on larger cohorts, the method’s sample throughput has to be further improved.

## Data Availability

The raw mass spectrometry proteomics data for the project have been deposited to the JPOSTrepo^54^ server JPOST ID (JPST002988) and PXID (PXD050708).

## Supporting Information Available

Supplementary methods; Figure S1, Fabrication of hemoglobin conjugated magnetic nanoparticles (Hb@MNPs); Figure S2, Representative SDS page image of enriched haptoglobin from serum sample; Figure S3, DIA parameter optimization; Figure S4, Venn diagram of overlapped glycopeptides generated in DDA, DIA, and hybrid DDA/DIA spectral library; Figure S5, Comparison of fragment similarity between DDA and DIA data set with Byonic scores in different ranges; Figure S6, score distribution of Byonic-based DIA analysis; Figure S7, examples of glyco-PSM spectra identified from Byonic; Figure S8, Venn diagram of overlapped Hp glycopeptide uniquely identified by DDA and DIA across all sera; Figure S9, Summary of top ten abundant glycoforms per N-site from HCC and HBV samples using the DIA method (PDF)
Table S1, Clinical characteristics of patients in this study (XLSX)
Table S2, Haptoglobin recovery from HBV and HCC samples using Hb@MNPs (XLSX)
Table S3, Spectral library of Hp glycopeptides generated from DDA, DIA, and hybrid DDA/DIA data sets from triplicate HCC serum samples (XLSX)
Table S4, Identified Gp using DDA and DIA (DDA, DIA, hybrid DDA/DIA library) from triplicate HCC serum samples (XLSX)
Table S5, 217 glycopeptides uniquely present in DIA using the spectral library from DIA data sets (XLSX)
Table S6, Spectral library of Hp glycopeptides generated from DIA data sets of personalized Hp glycopeptide identification across all patient samples (XLSX)
Table S7, Identified glycopeptides from HBV (*n* = 5) and HCC (*n* = 5) patients using the DDA method (XLSX)
Table S8, Identified glycopeptides from HBV (*n* = 5) and HCC (*n* = 5) patients using the DIA method (XLSX)
Table S9, 54 glycopeptides uniquely identified in HCC from the DIA method (XLSX)
Table S10, Site-specific N-glycopeptide differentially expressed in HCC compared to HBV samples (XLSX)

## Abbreviations

Hp: Haptoglobin
DDA: Data-Dependent Acquisition
DIA: Data-Independent Acquisition
MS: Mass Spectrometry
Hb: Hemoglobin
MNPs: Magnetic Nanoparticles
