# Supplementary material for: Data Independent Acquisition Mass Spectrometry Enhanced Personalized Glycosylation Profiling of Haptoglobin in Hepatocellular Carcinoma
Source: J Proteome Res. 2024 Jul 12;23(8):3571–84. doi: 10.1021/acs.jproteome.4c00227 (PMC11301664; doi:10.1021/acs.jproteome.4c00227)
Supplement: Supplementary file 1 — pr4c00227_si_001.pdf [file pr4c00227_si_001.pdf]

Data Independent Acquisition Mass Spectrometry Enhanced Personalized Glycosylation Profiling of Haptoglobin in Hepatocellular Carcinoma

Tiara Pradita[1,2,3,+], Yi-Ju Chen[1,+], Tung-Hung Su[4,5], Kun-Hao Chang[1,6,7], Pei-Jer Chen[4,5,8,9],

Yu-Ju Chen[1,2,10,].*

[1]Institute of Chemistry, Academia Sinica, Taipei 115, Taiwan

[2]Sustainable Chemical Science and Technology, Taiwan International Graduate Program, Academia Sinica, Taipei 115, Taiwan

[3]Department of Applied Chemistry, National Yang Ming Chiao Tung University, Hsinchu 300, Taiwan

[4]Division of Gastroenterology and Hepatology, Department of Internal Medicine, National Taiwan University Hospital, Taipei, Taiwan

[5]Hepatitis Research Center, National Taiwan University Hospital, Taipei, Taiwan.

[6]Molecular Science and Technology Program, Taiwan International Graduate Program, Academia Sinica, Taipei 115, Taiwan

[7]Department of Chemistry, National Tsing-Hua University, Hsinchu 300, Taiwan

[8]Graduate Institute of Clinical Medicine, National Taiwan University College of Medicine, Taipei, Taiwan

[9]Department of Medical Research, National Taiwan University Hospital, Taipei, Taiwan.

[10]Department of Chemistry, National Taiwan University, Taipei 106, Taiwan

+ Equal contribution

*Corresponding Author:

Yu-Ju Chen, Ph.D

E-mail: yujuchen@gate.sinica.edu.tw

Table of contents

Supplementary methods……………………………………………………………………………………………………iii

Supplementary Figures……………………………………………………………………………………………………iv

Figure S1. Fabrication of hemoglobin conjugated magnetic nanoparticles (Hb@MNPs)...........………………………………………………………………………………………..iv

Figure S2. Representative SDS page image of enriched Haptoglobin from serum sample. (A) enriched Haptoglobin from HCC patient for methodology test (B) enriched Haptoglobin from HBV (n=5) and HCC (n=5) patient………………………………………...v

Figure S3. DIA parameter optimization. Glycopeptide identification from standard Hp sample with different (A) isolation window and (B) HCD energy, respectively…vi

Figure S4. Venn diagram of overlapped glycopeptides generated in DDA, DIA and hybrid DDA/DIA spectral library…………………………………………………………………….vi

Figure S5. Comparison of fragment similarity between DDA and DIA dataset with Byonic™ scores different range: (A)<100, (B)from 100-200 and (C)>300.......................vii

Figure S6. The score distribution of Byonic™-based DIA analysis. (A) The number and proportion of spectra in the score range of <100, 100-150, 150-200, and > 200. (B) Categorizing the spectra with their identified glycan types in different score ranges. The percentage presents the proportion of each oligosaccharide type in the corresponding score range…………………………………………………………viii

Figure S7. The examples of glyco-PSM spectra identified from Byonic™ with (A) score <100, (B)score 100-50, (C) score 150-200, and (D) score >200………………………………...ix

Figure S8. Venn diagram of overlapped unique identified Hp glycopeptide between DDA and DIA across all serum……………………………………………………………………………..ix

Figure S9. Exploration analysis of top ten abundant glycoforms per N-site from HCC and HBV samples using DIA method. (A) N184 (B)N207 (C)N211 (D)N241…………………………………………………………………………………………………….x

Supplementary Table S1-Table S10……………………………………………………Separated excel files

Supplementary methods

LC-MS/MS Analysis

In DDA mode, the included charge states were 2 to 6 and the maximum injection time was 100 msec. Tandem MS was performed by isolating the precursor ions at a window of 2 Th in the quadrupole and fragmented by a product-dependent stepped higher-energy collisional dissociation (HCD) workflow. For MS2, first, HCD at a normalized lower-collision energy setting of 26 was acquired for the top-10 precursors detected in MS1, and detected in the Orbitrap at a resolution setting of 30,000, with a target value of 5×10^4 ion count and a maximum injection time of 25 msec. The second step was triggered when the diagnostic oxonium ions detected in the acquired HCD MSMS spectra were *m/z* 138.0545 for the HexNAc+ fragment; *m/z* 204.0867 for the HexNAc+ fragment; and/or *m/z* 366.1396 for the HexHexNAc+ fragment. The product-dependent stepped HCD fragmentation was set at 35 ± 8% collision energy (with three NCE 27%, 35%, and 43%) and detected simultaneously in the Orbitrap at a resolution setting of 30,000 with a target value of 5×10^4 ion count. The maximum injection time was 50 msec and the dynamic exclusion duration was set to 40 s with 10 ppm tolerance around the selected precursor and its isotopes. Monoisotopic precursor selection was turned on.

Supplementary figures

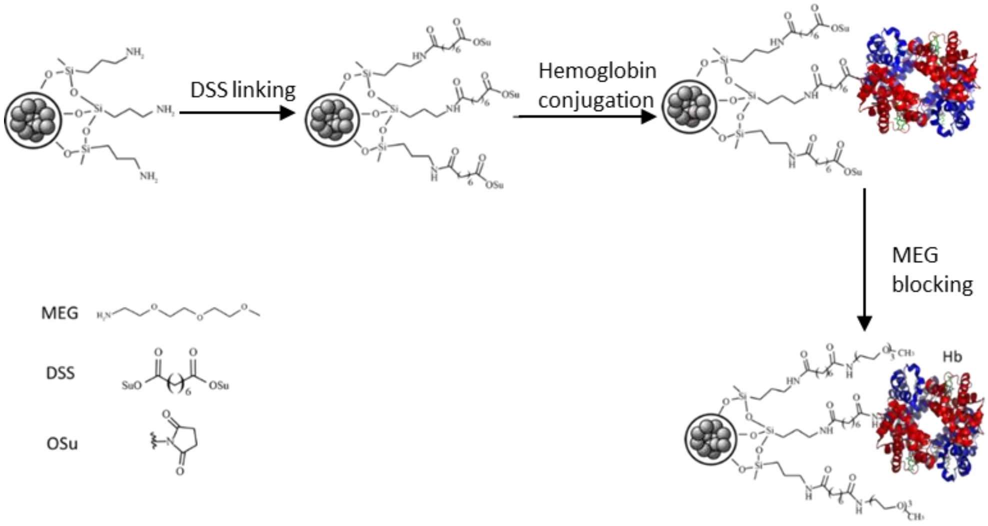

Figure S1. Fabrication of hemoglobin conjugated magnetic nanoparticles (Hb@MNPs). The Hb@MNPs was fabricated by firstly incubate DDS with NH_2-Fe_3O_4 core with the ratio of 5:1 (DSS:NH_2-Fe_3O_4 core) for 6 hours at room temperature, followed by the incubation of hemoglobin for 6 hours at 4°C. MEG was used for blocking unnecessary attachment to the particles.

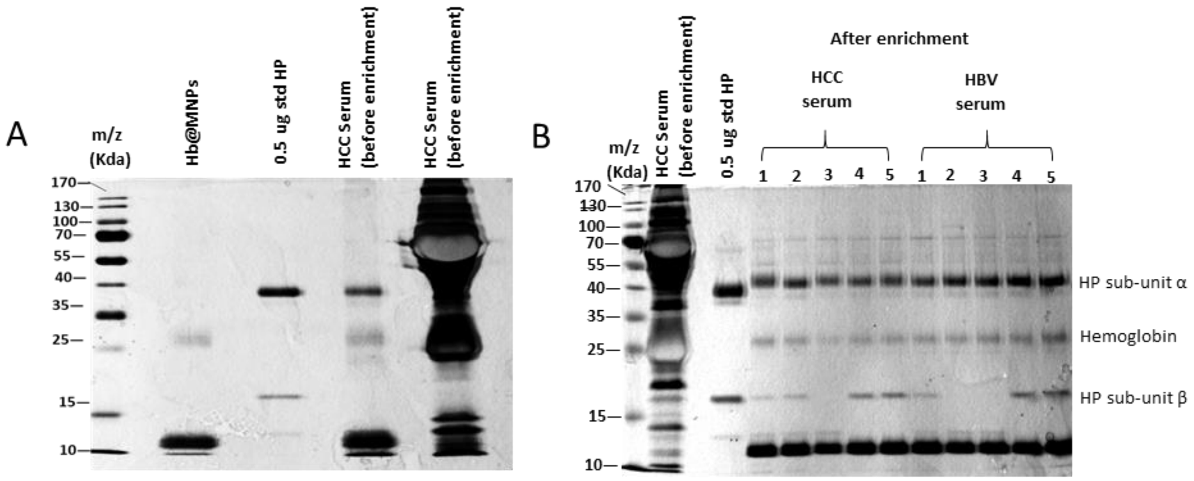

Figure S2. Representative SDS page image of enriched Haptoglobin from serum sample. (A) enriched Haptoglobin from HCC patient for methodology test (B) enriched Haptoglobin from HBV (*n=5*) and HCC (*n=5*) patient. Quality control of each patient serum was done by taking 10% (~0.5 µg of Hp) of the eluted haptoglobin and compared with 0.5 µg standard haptoglobin, quantitation of haptoglobin amount were determined using SDS-PAGE followed by silver stain. Utilizing image-J software, we calculated the %recovery of haptoglobin from serum sample.

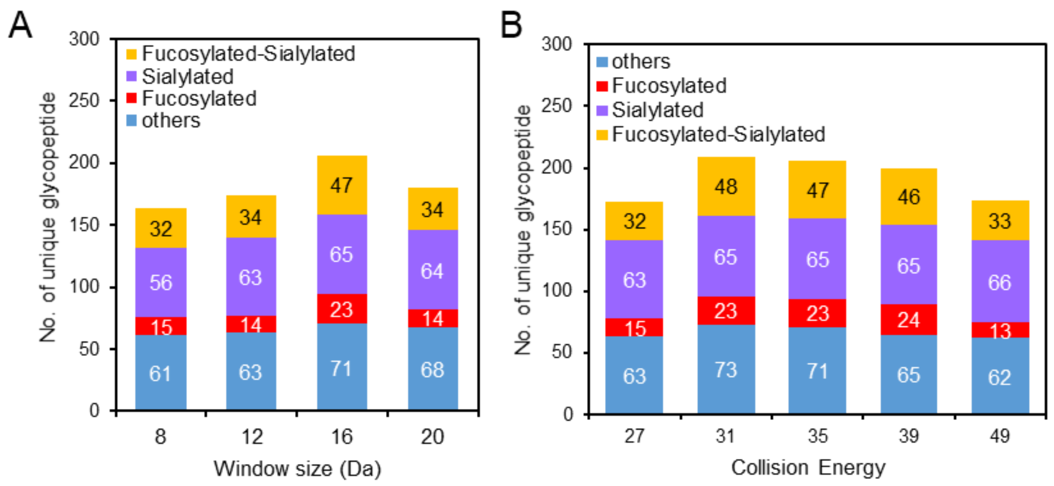

Figure S3. DIA parameter optimization. Glycopeptide identification from standard Hp sample with different (A) isolation window and (B) HCD energies, respectively.

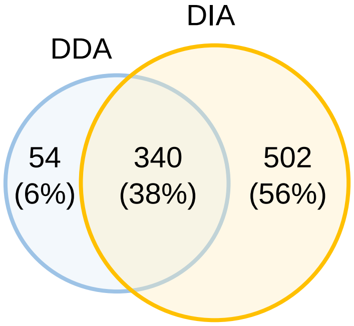

Figure S4. Venn diagram of overlapped glycopeptides generated in DDA, DIA and hybrid DDA/DIA spectral library.

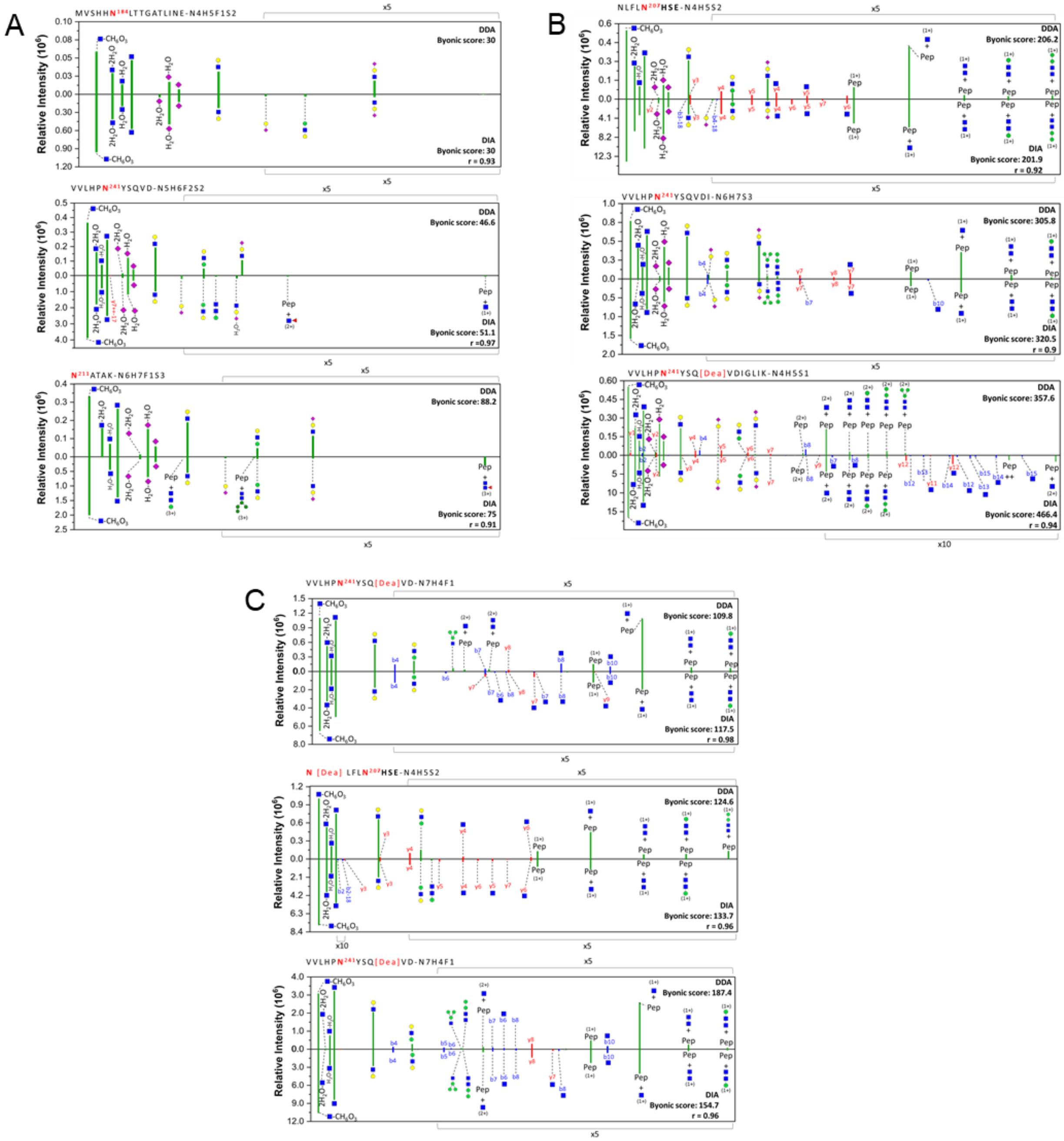

Figure S5. Comparison of fragment similarity between DDA and DIA dataset with Byonic™ scores different range: (A)<100, (B)from 100-200 and (C)>300.

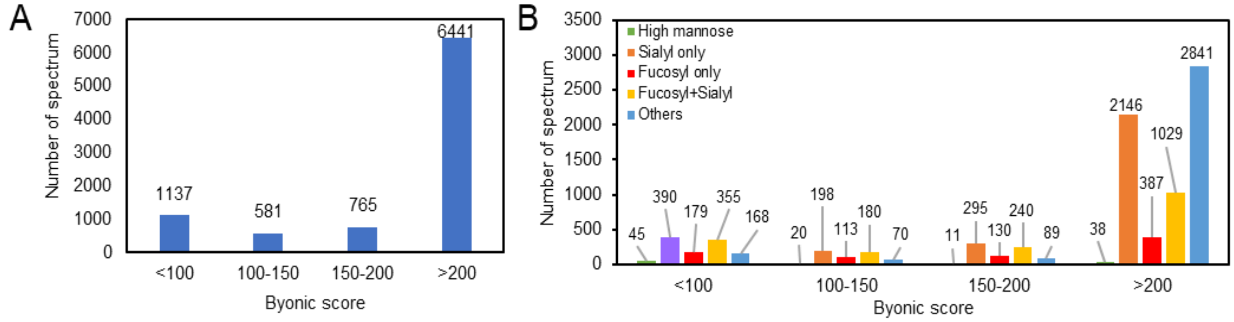

Figure S6. The Byonic™ score distribution of library-based DIA analysis. (A) The number and proportion of spectra in the score range of <100, 100-150, 150-200, and > 200. (B) Categorizing the spectra with their identified glycan types in different score ranges. The number indicates of number of each oligosaccharide type in the corresponding score range.

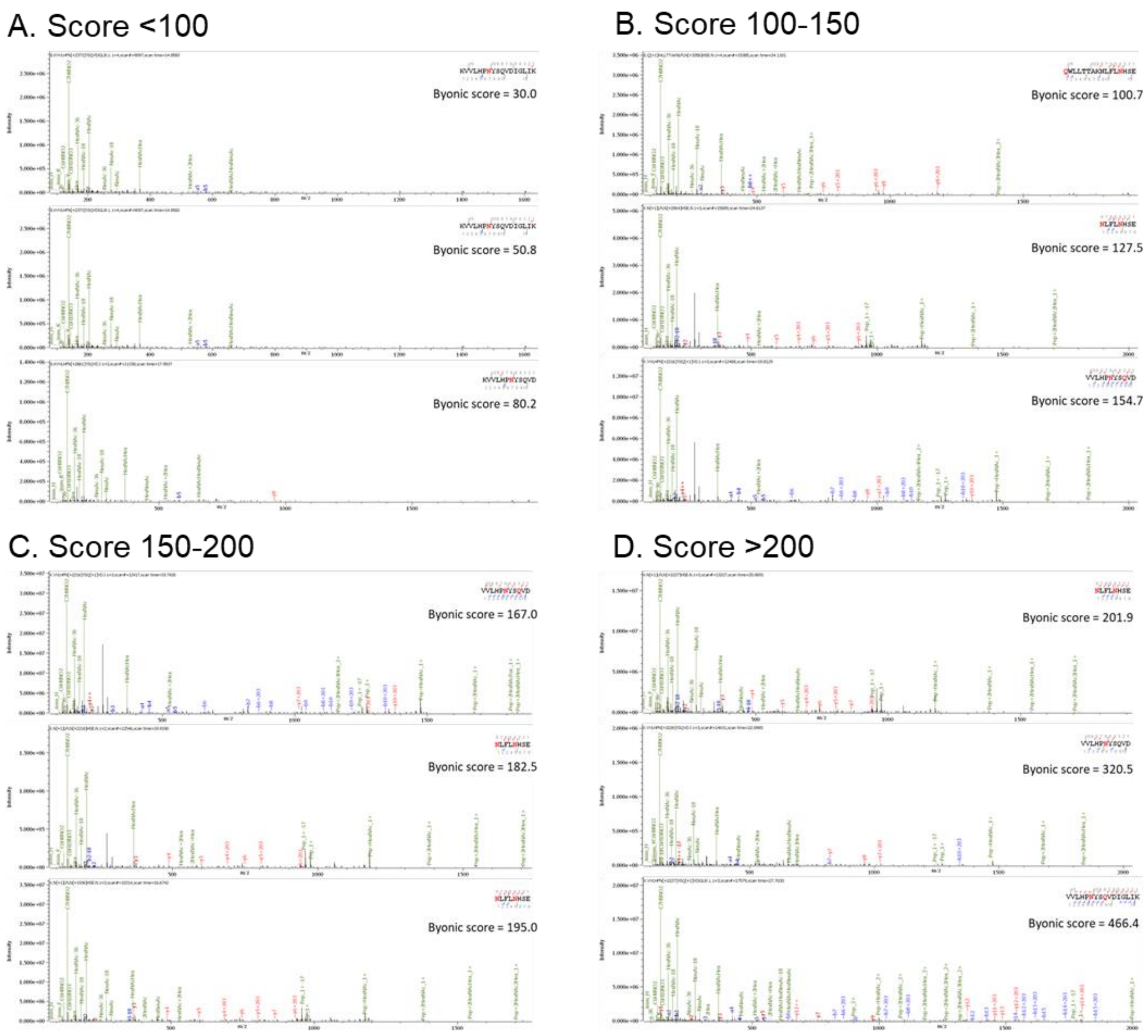

Figure S7. The examples of glyco-PSM spectra identified from Byonic™ with (A) score <100, (B)score 100-50, (C) score 150-200, and (D) score >200.

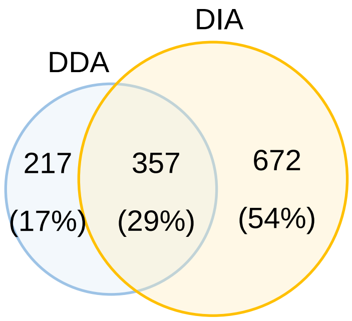

Figure S8. Venn diagram of overlapped unique identified Hp glycopeptide between DDA and DIA across all serum.

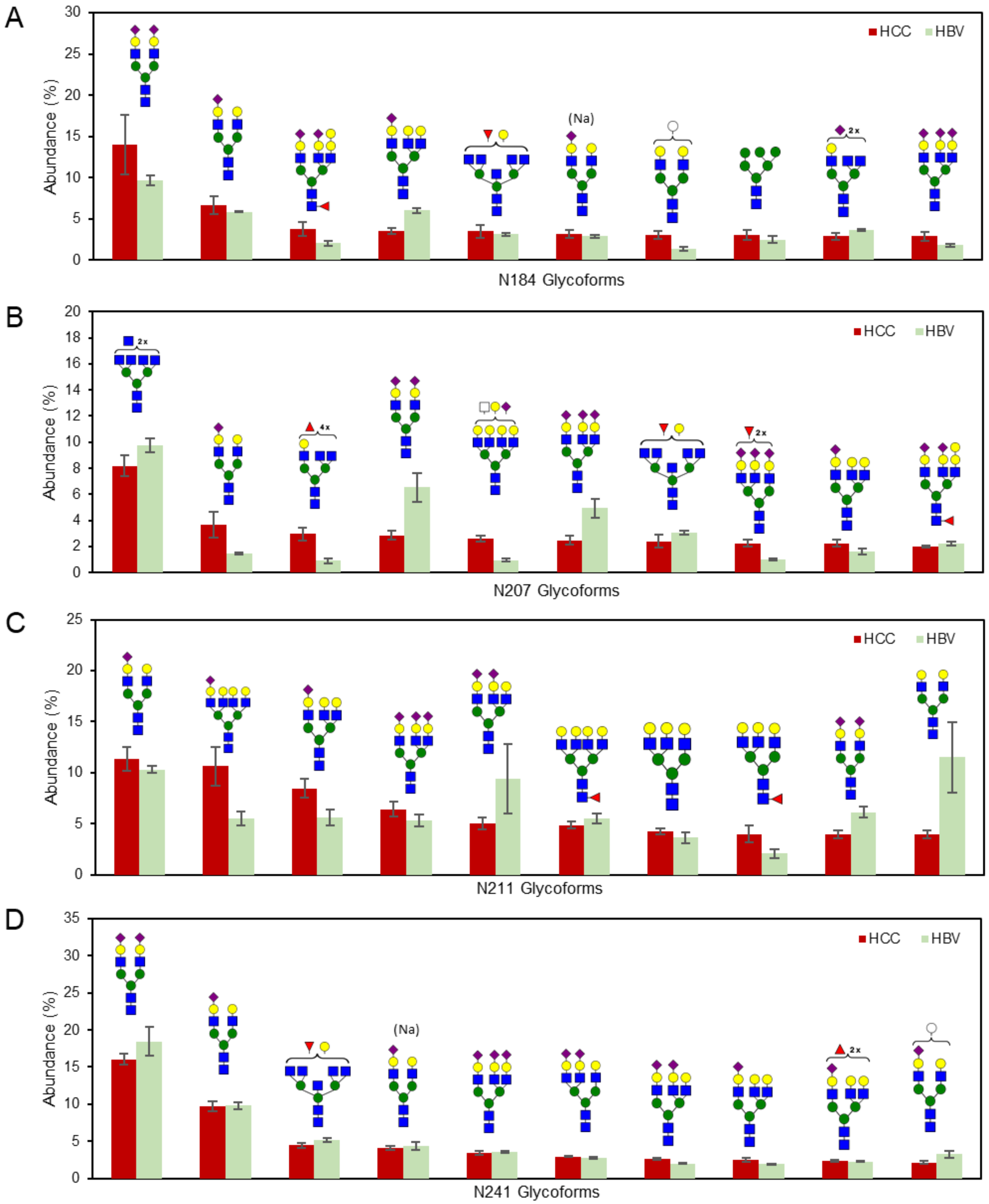

Figure S9. Exploration analysis of top ten abundant glycoforms per N-site from HCC and HBV samples using DIA method. (A) N184 (B)N207 (C)N211 (D)N241. (Na): monosodium residue attached to the glycan
